# Asymmetric dominance and asymmetric mate choice oppose premating isolation after allopatric divergence

**DOI:** 10.1002/ece3.1372

**Published:** 2015-03-13

**Authors:** Kristina M Sefc, Caroline M Hermann, Bernd Steinwender, Hanna Brindl, Holger Zimmermann, Karin Mattersdorfer, Lisbeth Postl, Lawrence Makasa, Christian Sturmbauer, Stephan Koblmüller

**Affiliations:** 1 Institute of Zoology, University of GrazUniversitätsplatz 2, 8010, Graz, Austria; 2 Department of Fisheries, Lake Tanganyika Research UnitPO Box 55, Mpulungu, Zambia

**Keywords:** Assortative mating, Cichlidae, male–male competition, secondary contact, speciation, *Tropheus*

## Abstract

Assortative mating promotes reproductive isolation and allows allopatric speciation processes to continue in secondary contact. As mating patterns are determined by mate preferences and intrasexual competition, we investigated male–male competition and behavioral isolation in simulated secondary contact among allopatric populations. Three allopatric color morphs of the cichlid fish *Tropheus* were tested against each other. Dyadic male–male contests revealed dominance of red males over bluish and yellow-blotch males. Reproductive isolation in the presence of male–male competition was assessed from genetic parentage in experimental ponds and was highly asymmetric among pairs of color morphs. Red females mated only with red males, whereas the other females performed variable degrees of heteromorphic mating. Discrepancies between mating patterns in ponds and female preferences in a competition-free, two-way choice paradigm suggested that the dominance of red males interfered with positive assortative mating of females of the subordinate morphs and provoked asymmetric hybridization. Between the nonred morphs, a significant excess of negative assortative mating by yellow-blotch females with bluish males did not coincide with asymmetric dominance among males. Hence, both negative assortative mating preferences and interference of male–male competition with positive assortative preferences forestall premating isolation, the latter especially in environments unsupportive of competition-driven spatial segregation.

## Introduction

Speciation depends on the reduction of gene flow between diverging taxa. In allopatric populations, reproductive isolation is readily initiated by physical barriers, before, eventually, intrinsic barriers to gene flow complete speciation (Coyne and Orr 2004). The evolution of behavioral isolation, for instance by assortative mating, is an important component of this process (Stelkens et al. 2009). Mate preferences have been shown to have a genetic basis in many animals (reviewed by Bakker and Pomiankowski 1995; within species: Jennions and Petrie 1997; among species: Laturney and Moehring 2012), and there is a growing evidence for the importance of sexual imprinting during early development. Experiments demonstrated that young birds, mammals, and fish cross-fostered to a related (sub)species develop sexual preferences for the foster mother's (sub)species (e.g., Clayton 1990; Kendrick et al. 1998; Verzijden et al. 2008). In most natural rearing environments, young animals are likely to imprint on conspecific signals, and learnt preferences may therefore sustain sexual isolation between closely related taxa (Sorenson et al. 2003; Kozak et al. 2011; Verzijden et al. 2012).

Mate choice is also influenced by intrasexual competition, and inter- and intrasexual selection may reinforce or oppose each other (reviewed e.g., by Qvarnström and Forsgren 1998; Wong and Candolin 2005; Hunt et al. 2009). For example, in various animal species ranging from crickets to pheasants, in which mating success is skewed toward dominant males, females display no preferences for dominant males when tested in the absence of male–male competition (Forsgren 1997; Reichard et al. 2005; Spence and Smith 2006; Casalini et al. 2009; and more examples in Qvarnström and Forsgren 1998). In contrast to numerous studies on intra- and intersexual selection pressures within populations, empirical evidence regarding how and to what degree interactions between mate preferences and intrasexual competition affect assortative mating and speciation processes is limited (Qvarnström et al. 2012). In resource-based breeding systems, for instance, interspecific competition can on the one hand lead to habitat segregation and hence strengthen premating isolation (Vallin and Qvarnström 2011), but can on the other hand interfere with assortative mating by constraining the choice of conspecific males. The latter situation arises when the outcome of male–male competition determines the availability of potential mates. A consistent competitive advantage of one species or population over the other can then provoke asymmetric hybridization (Rosenfield and Kodric-Brown 2003; Bierbach et al. 2012; Grava et al. 2012a; Vallin et al. 2012a).

Differences in competitive abilities between species or populations can be associated with body size (Jones et al. 2008) or with traits signaling competitive ability such as vocalization (Grava et al. 2012b; Pasch et al. 2013) and body coloration (Dijkstra et al. 2005). Carotenoid-based coloration is frequently correlated with competitive success in intraspecific contests (e.g., sticklebacks, Bakker and Sevenster 1983; cichlids, Evans and Norris 1996; widowbirds, Pryke et al. 2001; Ninnes and Andersson 2014; mandrills, Setchell and Jean Wickings 2005; lizards, Hamilton et al. 2013). In polymorphic populations, red-colored morphs are known to dominate over other morphs (cichlids, Barlow 1983; finches, Pryke and Griffith 2006; lizards, Healey et al. 2007; but see Sacchi et al. 2009), and red male dominance was also observed in competition between sympatric species (Dijkstra et al. 2005). Dominance of red males may reflect the dependency of carotenoid signaling on body condition and androgen levels, which could entail covariance of redness with aggressiveness and competitive ability (Sinervo et al. 2000; Pryke and Griffith 2006; Dijkstra et al. 2009). Moreover, red color may directly intimidate contest opponents (Rowland et al. 1995). Masking the red coloration of naturally red individuals, for example, by painting or in monochromatic light, frequently erases their competitive advantage (Evans and Norris 1996; Dijkstra et al. 2005; Healey et al. 2007; Pryke 2009). If body coloration can also affect outcomes of competition between differently colored contestants which have no history of sympatry, this could have a bearing on reproductive isolation and hence speciation processes when allopatric color variants come into secondary contact.

Cycles of population fragmentation and secondary contact fuel the diversification of cichlid fishes in the rocky littoral of the East African Great Lakes (Rossiter 1995; Genner and Turner 2005). For stenotopic species restricted to rocky habitat, small habitat barriers such as sandy bays or river estuaries suffice to restrict gene flow and allow for microallopatric differentiation (Genner and Turner 2005; Koblmüller et al. 2008). As long as the external barriers persist, genetic and phenotypic differentiation can proceed without much gene flow, which allowed many rock-dwelling species to diversify into geographic color variants or sister species. However, lake-level fluctuations, which occur in timescales of tens to hundreds of thousands of years (Cohen et al. 1997; Scholz et al. 2003; McGlue et al. 2008), alter the shoreline structure and entail secondary contact among differentiated populations. The degree of reproductive isolation built up in allopatry has a large effect on the impact of secondary contact on the speciation process. Mate choice experiments demonstrated that allopatric color pattern differentiation among cichlid populations and species is sometimes, but not as a rule, associated with assortative mate preferences (Table1 in Maan and Sefc 2013). Both genetic factors (Haesler and Seehausen 2005) and sexual imprinting on parental phenotypes (Verzijden and ten Cate 2007) have been implicated in cichlid mate choice. Importantly, sexual imprinting can generate assortative preferences virtually as a by-product of phenotypic differentiation in allopatry and can therefore promote behavioral isolation in secondary contact (Irwin and Price 1999; Verzijden et al. 2012).

**Table 1:** Summary of female preferences, male dominance, and assortative mating in mate choice, contest, and pond mating experiments

	2-way mate choice (excluding male–male competition)	Male–male competition	Pond mating	Proposed effect of color-dependent male–male competition (MMC) on assortative mating
	% females with positive assortative preferences, inference on female preferences (*P* for random preferences)	% contests won, inference on dominance (*P* for even odds)	% assortative matings, inference on mate choice (*P* for random mating)	
Red versus bluish morph *Tropheus* (R-B)				
Red morph	100%, strongly pos. assort. (*P* = 0.008)^1^	96%, dominant (*P* = 1.22 × 10^−5^)	100%, strongly pos. assort. (*P* = 1.49 × 10^−8^)	Both MMC and female preferences promote positive assortative mating
Bluish morph	86%, weakly pos. assort. (*P* = 0.06)^1^	4%, subordinate (*P* = 1.22 × 10^−5^)	65%, random (*P* = 0.149)	MMC interferes with positive assortative female preferences
Red versus yellow-blotch morph *Tropheus* (R-YB)				
Red morph	Not investigated	77%, dominant (*P* = 0.025)	100%, pos. assort. (*P* = 0.0002)	MMC and female preferences favor positive assortative mating
Yellow-blotch morph	Not investigated	23%, subordinate (*P* = 0.025)	60%, random (*P* = 0.53)	MMC promotes negative assortative mating
Yellow-blotch versus bluish morph *Tropheus* (YB-B)				
Yellow-blotch morph	42%, random (*P* = 0.58)^2^	45%, (*P* = 0.78)	18%, neg. assort. (*P* = 0.0007)	Excess of negative assortative mating independent of MMC
Bluish morph	91%, pos. assort. (*P* = 0.006)^2^	55%, (*P* = 0.78)	88%, pos. assort. (*P* = 1.22 × 10^−6^)	Positive assortative mating independent of MMC

pos. assort., positive assortative; neg. assort., negative assortative.

1 Data from Egger et al. (2008).

2 Data from Egger et al. (2010).

In the resource-based and lek-like mating systems of many cichlid species, territories, nest sites, or display sites defended by males play a critical role in determining male mating success (McKaye et al. 1990; Genner et al. 2008; Schaedelin and Taborsky 2010; Hermann et al. 2015). Given that experimental manipulation of territory characteristics can override conspecific female preferences (Dijkstra et al. 2008), the strength of behavioral isolation in secondary contact may depend on how the competitive abilities of differentiated taxa interact with their sexual preferences.

**Figure 1:**
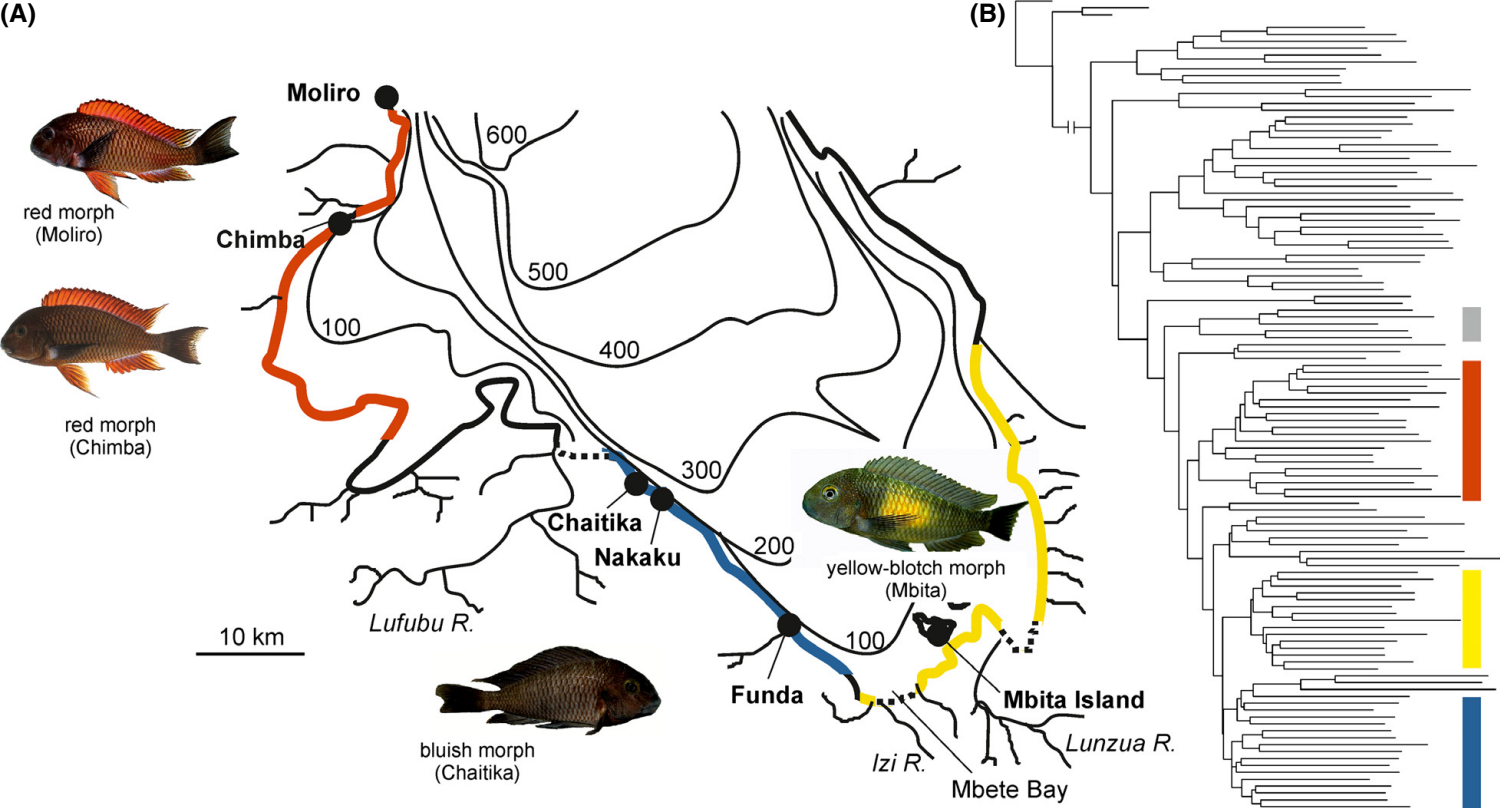
*Tropheus* color morphs used in this study. (A) The distribution of the red, bluish, and yellow-blotch morphs in southern Lake Tanganyika is represented by the colored shoreline. Uncolored shore sections are inhabited by populations carrying genetic signatures of introgression between the adjacent color morphs. Three major dispersal barriers formed by the estuaries of the Lufubu, Izi, and Lunzua are indicated by hatched lines. Photographs by Wolfgang Gessl (Moliro, Chimba), Peter Berger (Mbita), and C.M.H. (Chaitika). (B) Genetic relationships among the tested color morphs in an AFLP marker-based tree representing *Tropheus* spp. populations sampled around Lake Tanganyika (Egger et al. 2007). Colored bars mark clades containing red, yellow-blotch, and bluish morph *Tropheus*. The gray bar indicates the clade of Murago-type *Tropheus* (see Discussion).

This study addresses the impact of male–male competition on behavioral isolation in simulated secondary contact. We tested for asymmetries in competitive abilities among allopatric populations of the cichlid fish *Tropheus* and compared assortative mating in the presence of male–male competition to expectations based on female preferences in previous two-way choice experiments (Egger et al. 2008, 2010). *Tropheus* are typical stenotopic rock-dwelling cichlids of the shallow littoral in Lake Tanganyika, with rich geographic color pattern variation among closely related populations and a history of population fragmentation and secondary contact driven by lake-level fluctuations (Sturmbauer et al. 2005; Egger et al. 2007; Koblmüller et al. 2011). Within populations, the mating success of males depends on their territories (Yanagisawa and Nishida 1991; Hermann et al. 2015), which raises the possibility that competition for territories among males of differentiated color morphs affects patterns of assortative mating in secondary contact. Three color morphs were tested against each other (Fig.1). To test whether male competitive abilities differ between morphs, we staged dyadic contests for territories between differently colored males. To assess reproductive isolation among color morphs in the presence of male–male competition, we stocked experimental ponds with multiple males and females of two *Tropheus* color morphs per pond and genotyped offspring to determine mating patterns.

## Material and Methods

### Information on the study species and female preferences in two-way choice experiments

The genus *Tropheus* is endemic to Lake Tanganyika, Africa. Solitary females defend their own feeding territories, but bond temporarily with their mates for several days to weeks prior to spawning. While paired, females feed intensively on epilithic algae in their mate's territory, which allows them to mature for spawning (Yanagisawa and Nishida 1991). After spawning, females abandon their mate for maternal mouthbrooding (Yanagisawa and Nishida 1991). Territory quality influences male mating success (Yanagisawa and Nishida 1991; Hermann et al. 2015), and competition for territories ensues from the high density of *Tropheus* in the rocky littoral of Lake Tanganyika (e.g., Sturmbauer et al. 2008).

The classification of the numerous different geographic color variants into species is ambiguous (Egger et al. 2007; Konings 2013), which is why populations are typically identified by locality and color pattern type rather than by species names. Gradual among-population differences in color patterns generally increase with geographic distance between populations, and major habitat barriers often separate highly distinct color morphs. In *Tropheus*, a “color morph” defines closely related populations with similar color pattern. The three color morphs used in this study (red morph, bluish morph, and yellow-blotch morph) inhabit different stretches of shoreline in southern Lake Tanganyika, are closely related to each other, and are currently separated by river estuaries (Fig.1; Egger et al. 2007). The bluish and the yellow-blotch morphs differ mainly in the presence of a yellow blotch at the flank on a dark bluish-gray background, and the red morph is characterized by red to orange body and fin coloration. There is no sexual dimorphism in color patterns or body size in these populations. The yellow-blotch and bluish *Tropheus* have been assigned to *T. moorii*, whereas the red morph has been proposed to represent a different, not formally described, species (Konings 2013; see Discussion). In previous two-way female choice experiments (red vs. bluish morph; bluish vs. yellow-blotch morph), all red females preferred red males over bluish males, and most bluish females preferred bluish males over red and yellow-blotch males (Table1). In contrast, most yellow-blotch females courted either preferentially with the bluish males or equally with both homomorphic and bluish males, the latter making it impossible to determine a preference for one morph over the other (Egger et al. 2008, 2010). Males of the red morph discriminate against females of distinct populations (Zoppoth et al. 2013). Preferences in these two-way choice experiments were inferred from courtship behavior (and occasionally from spawning during the experiment) in full contact between the male and female test fish, allowing visual, olfactory, and acoustic communication. Females were given access to only one of the two stimulus males at a time, and there were no interactions between the males (Egger et al. 2008).

### Male–male competition experiments

Pairwise encounters between males were staged to test for color morph-dependent advantages in competition for territories. Yellow-blotch versus bluish males (YB-B; wild-caught individuals of populations Mbita and Funda, respectively; Figure1) and red versus yellow-blotch males (R-YB; wild-caught individuals of populations Chimba and Mbita, respectively; Figure1) were tested in concrete ponds (100 × 80 × 80 cm) furnished with a single rock cairn (approx. height 50 cm, diameter 50 cm). The ponds were situated in the Rivatrop fish breeding facility on the southern shore of Lake Tanganyika near Mpulungu, Zambia, and were supplied with lake water. The males had previously been used in the mating experiments described below. Only males from different mating ponds competed against each other. Prior to the trials, males were kept individually in nursery cages (16.5 × 14 × 12.5 cm) floating within their previous mate choice ponds, their weight was taken with a spring scale, and their standard length was measured. In the test pond, the two competitors were released simultaneously at opposite sides of the rock cairn. The fish were observed until one of them had defended the rock cairn against the opponent for 5 min and was declared the winner of the contest. If no winner emerged, most often because both fish settled in the rock cairn, the trial was terminated after 90 min (YB-B) or 30 min (R-YB). The duration of R-YB trials was reduced from 90 to 30 min because in the YB-B trials, contests were either settled within less than 30 min or remained tied for the remaining observation time. All possible combinations of yellow-blotch versus bluish males (92 pairs) were tested and yielded 84 settled encounters. In the R-YB experiment, 41 pairs were roughly matched by weight, with a maximum difference of 5.5 g, and yielded 31 settled encounters.

Red and bluish males (R-B; red males from population Moliro; bluish males: six from Nakaku, one from Chaitika; Figure1) were tested in tanks (150 × 70 × 50 cm) at our department (Univ. Graz, Austria). Test fish were wild-caught adults imported by an ornamental fish trader. Initially, a similar procedure was used as described above for the contests staged in ponds, that is releasing the contestants simultaneously in a test tank, in which a 2-bottle wine rack served as territory focus. As the red males always took possession of the rack immediately and the bluish males made no attempts toward the rack, the design was changed to encourage interaction between opponents by allowing the bluish male to settle in the test tank before competing with the red male. The test tank was divided into two equally sized compartments by a mesh partition (13 mm grid size), and 2-bottle wine racks (23 × 25 × 12.5 cm) were positioned next to the partition in each compartment. This resulted in a single central territory divided into two halves by the partition (Fig. S1). The red and the blue male were then introduced into the separate compartments. Morphs alternated between right and left compartments between trials. After an acclimation period of 24 h, the partition was removed, and the interaction between the pair was observed. Contests for territories were settled on average within 4 min (maximum <10 min), when the dominance relationships between the contestants were clearly evident by coloration and behavior, upon which the fish were immediately separated. All possible pairs of seven red males and seven bluish males were tested. Fish were weighed with a spring scale prior to each trial.

To test whether males of a particular morph were equally likely to lose or win against their opponent morph, logistic regressions (GLMM with a binomial error distribution and a logit link; R package lme4, Bates and Maechler 2009) were calculated with outcome as binary response variable (winning/losing of focus morph), relative size (in YB-B and R-YB) or weight difference (in R-B) as fixed factor, and male identities as random factors. Relative size or weight differences (RSD and RWD, respectively) were calculated as size (weight) difference between opponents divided by the sum of size (weight) of both opponents. Size and weight of the fish are closely correlated and both can be used to represent body size differences. Model intercepts deviating significantly from zero indicated higher or lower odds of winning for the focal morph.

### Pond mating experiments

Three pond experiments addressed reproductive isolation between yellow-blotch and bluish *Tropheus* (YB-B), between red and bluish *Tropheus* (R-B), and between red and yellow-blotch *Tropheus* (R-YB; Fig.1). For each experiment, five (YB-B and R-B) or four (R-YB) replicate ponds were stocked with eight females and four males of each of the two morphs considered in the experiment (24 fish per pond). In experiment YB-B, the yellow-blotch and bluish morphs were represented by populations at Mbita Island and Funda, respectively. For experiment R-B, red and bluish morph fish were collected from Chimba and Chaitika, respectively. For experiment R-YB, red and yellow-blotch morph fish originated from Chimba and Mbita Island, respectively (Fig.1). The test fish were wild-caught adults, which had grown up in their respective populations. Therefore, all test fish had had the same opportunities to imprint on their parental populations.

The mate choice ponds were situated in the Rivatrop fish breeding facility at Lake Tanganyika. The concrete ponds were supplied with lake water. Water changes occurred daily, and fish were fed with cassava mash and commercial fish food. Pond dimensions were 2.5 × 0.9 × 0.8 m (length × width × depth). Eight rock cairns, built of large rocks collected from the lake, were distributed evenly in each pond and provided shelter and territorial centers for the fish.

Fin clips were taken for Chelex DNA extraction (Walsh et al. 1991) from the adults both at the time of stocking and when offspring were collected, which was after 1 year in experiments YB-B and R-B. In experiment R-YB, offspring were collected already after 6 months for logistic reasons. Several of the fish which had died during the time of the experiment had left offspring. In all ponds, at least one male and female of each morph had survived throughout the experiment (see Tables S1–S3). Fin clips were also taken from all collected offspring for genetic parentage analysis. The numbers of genotyped offspring per pond were 100, 101, 101, 97, 135, respectively, in the five ponds of the YB-B experiment; 98, 101, 91, 88, 87, respectively, in the R-B experiment; and 75, 25, 37, 40 in the R-YB experiment.

Parentage assignment was based on genotypes at four to eight microsatellite loci. First, all individuals were genotyped at markers TmoM11, TmoM27 (Zardoya et al. 1996), UME002, and UME003 (Parker and Kornfield 1996). Tests for Hardy–Weinberg equilibrium were conducted in Arlequin v.3.0 (Excoffier et al. 2005) using the stocked adults as population samples, and no deviations from equilibrium were detected. The software CERVUS was used for identifying parent–offspring triads within each pond (Marshall et al. 1998). Parentage was considered resolved when a juvenile was assigned to a single parent pair (i.e., one male and one female) without allelic mismatch. When multiple candidate parent pairs remained, the involved individuals were genotyped at three to four additional loci from the following set: Ppun9 (Taylor et al. 2002), Pmv3, Pmv17 (Crispo et al. 2007), Hchi6 (Maeda et al. 2007), UNH130, UNH154 (Lee and Kocher 1996), and Pzeb3 (van Oppen et al. 1997). Additional genotyping always resulted in the exclusion of all but one parent pair. Numbers of offspring per mated pair are given in the supplementary Tables (S1–S3). PCRs, sizing of alleles against an internal size standard (GeneScan-500 ROX) on an ABI 3130xl sequencer (Applied Biosystems, Vienna, Austria), and allele scoring in the Genemapper vs. 3.7 software, were carried out as previously described (Koch et al. 2008).

Mating patterns of females were inferred from parentage data by conservatively assuming that each batch of full sibs assigned to a particular parent pair corresponded to a single mating event. Clutch sizes of mouthbrooding yellow-blotch females captured in the lake ranged from 5 to 13 offspring (Egger et al. 2006), whereas higher fecundity, particularly of red females, was observed in the laboratory and is compatible with the largest brood sizes reconstructed from the pond data. As wild-caught *Tropheus* broods were found to be sired by a single male each (Egger et al. 2006), we considered maternal half sibs to represent different broods (see also Discussion).

Each reconstructed mating was scored as either positive or negative assortative. Because some of the stocked adults had died during the time of the experiment, we tested whether the numbers of surviving males and females correlated with the numbers of reconstructed matings. Data points were scored for females of the same population in the same pond, and no correlations were detected between (1) the number of reconstructed matings and the number of surviving females, (2) the number of positive assortative matings by females and the number of surviving same-population males, (3) the number of negative assortative matings by females and the number of surviving foreign-population males, and (4) the proportion of positive assortative matings by females and the proportion of same-population males among surviving males. Therefore, we concluded that the loss of some adults had not biased the observed proportions of positive and negative assortative matingsand continued with the analysis of the reconstructed matings.

In almost all ponds, some females had mated repeatedly (with different males), and these data points are not independent. Beyond that, any matings within a pond may not be independent of each other. The problem of potential dependency could be resolved in generalized linear mixed models (GLMM) by including female identity and pond as random factors. However, our data included several incidences of perfect prediction, that is, when all of the females in a pond scored either positive or negative assortative, which interferes with parameter estimation in GLMM (Heinze and Schemper 2002). An appropriate correction for the bias introduced by perfect prediction has not yet been developed for mixed models (G. Heinze, pers. comm.). Therefore, the following steps were taken to test the null hypothesis that females of a morph were equally likely to mate with homomorphic and heteromorphic males. (1) Mating data were analyzed for each pond separately as well as (2) pooled across all ponds within an experiment, in the latter case ignoring the pond-nested structure of the data. GLMMs with binomial error distributions and female identity as random factor were employed, if the data were not affected by perfect prediction, and two-tailed binomial tests were used in cases of perfect prediction. To avoid the problem of pseudoreplication originating from multiple matings of the same females in the binomial tests, each mated female was represented as only one single data point. (3) One-tailed probability values obtained from GLMM and binomial analyses of individual ponds within an experiment were combined using the weighted Z-method (Whitlock 2005). As fish grew at different rates during the experiment (Table S2) and their size at the time of mating is unknown, body size was not included in the analysis.

Differences in the odds of positive assortative mating between yellow-blotch and bluish *Tropheus* females in experiment YB-B were tested by including female morph as a fixed factor in the GLMM. This was not possible in the analysis of experiments R-B and R-YB due to perfect prediction of mating type by morph (positive assortative mating by all red females). GLMMs were calculated with the R package lme4 (Bates and Maechler 2009).

## Results

### Asymmetries in competitive abilities and asymmetric hybridization

#### Red versus bluish Tropheus (R-B)

*Male–male competition*. Red males won the territory in 47 of 49 settled trials (Table S1; odds in favor of winning in GLMM = 23.5 : 1, *P* = 1.23 × 10^−5^). Weight differences between opponent males did not affect the outcome of the contests (effect of RWD in GLMM: *β *= −1.6, *P* = 0.82).

**Figure 2:**
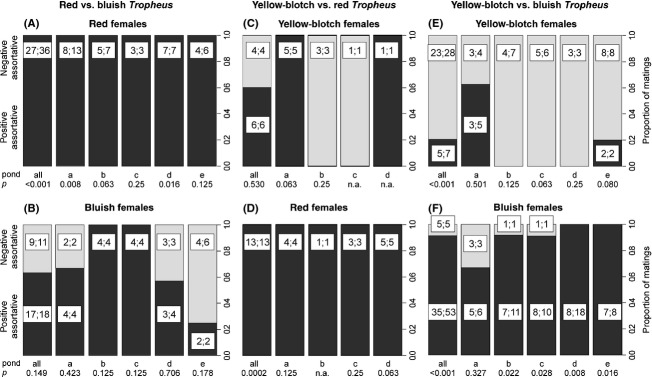
Proportions of positive and negative assortative mating by females in ponds stocked with red and bluish *Tropheus* (left-hand panels A and B), with red and yellow-blotch *Tropheus* (center panels C and D), and with yellow-blotch and bluish *Tropheus* (right-hand panels E and F). Within each panel, the left-hand bar represents matings summed across all ponds of the experiment, and the remaining bars report results for each pond separately. Insets in bars give the number of females and the number of matings in each mating type as (females;matings), which differ when certain females mated more than once. *P*-values underneath *x*-axis pond labels are the probabilities for the null hypothesis that negative and positive matings are equally likely and were obtained by binomial tests, when only one mating type was scored, and by linear models, when both mating types occurred.

*Mating*. Red females mated exclusively with red males. The deviation from random choice was significant both when ponds were analyzed together (27 females; two-tailed binomial test, *P* = 1.49 × 10^−8^) and separately (combined probabilities of one-tailed binomial tests, *P* = 4 × 10^−6^; Fig.2A).

For bluish females, the GLMM of mating data pooled across ponds yielded a 1.73 times higher probability to mate with bluish than with red males, but the deviation from random choice was not significant (*P* = 0.149). The proportions of mating types varied among ponds (Fig.2B). When ponds were analyzed separately and *P*-values combined across analyses, results were consistent with the pooled analysis, as positive assortative matings did not occur more frequently than expected by chance (combined *P*-values of one-tailed tests, *P* = 0.256). Three of the bluish females mated with males of both morphs (Table S2).

#### Red versus yellow-blotch Tropheus (R-YB)

*Male–male competition*. Red males won the territory in the majority of the settled trials (24 wins, 7 losses, Table S3; odds of 5.4 : 1 in favor of winning in GLMM, *P* = 0.025). Contest outcome was not affected by size differences between opponents (effect of RSD in GLMM: *β *= 9.2, *P* = 0.67).

*Mating*. Being run for a shorter period of time, this experiment yielded fewer offspring and fewer reconstructed matings than experiments R-B and YB-B (Fig.2B, Table S4). Red females mated exclusively with red males. The deviation from random choice was significant both when ponds were analyzed together (13 females; binomial test, *P* = 0.0002) and in the combination of separate analyses of ponds a, c, and d (combined probabilities of one-tailed binomial tests, *P* = 0.0038; Fig.2B). In pond b, only one of the red females was assigned offspring (Table S4).

For yellow-blotch females, the GLM of mating data pooled across ponds showed no deviation from random mating (odds of 1.5 : 1 in favor of positive assortative mating, *P* = 0.53). The small number of mated females per pond precluded the analyses of individual ponds.

### Asymmetric hybridization without color morph-dependent dominance

#### Yellow-blotch versus bluish Tropheus (YB-B)

*Male–male competition*. The YB-B competition trials detected no effect of color morph on male dominance relationships. Blue males won the territory in 46 trials, and yellow-blotch males won in 38 trials (Table S5). A GLMM estimated the odds in favor of winning for the blue males as 1.3 : 1 (*P* = 0.78). The relative size difference between males had a significant effect on the probability of winning a contest (effect of RSD: *β *= 50.5, *P* = 5.46 × 10^−5^).

*Mating*. Both bluish and yellow-blotch females were more likely to mate with bluish males. With mating data pooled across ponds, the GLMM estimated the odds in favor of mating with bluish males as 18.96 : 1 for bluish females (*P* = 1.22 × 10^−6^) and 6.51 : 1 for yellow-blotch females (*P* = 0.0007). Females of the two morphs differed significantly in their likelihood to mate with males of their own morph (*P* = 4.54 × 10^−9^). When ponds were analyzed individually (Fig.2C) and *P*-values combined, results were consistent with the analysis of pooled data, as both yellow-blotch and bluish females mated more frequently with bluish males than expected if choice was random (combined *P*-values of one-tailed tests, yellow-blotch: *P* = 0.023; bluish: *P* = 2 × 10^−6^). Three of the yellow-blotch females and five of the bluish females mated with males of both morphs (Table S6).

## Discussion

### Red male dominance and reproductive isolation

Color morph-dependent asymmetries in competitive abilities were described in polychromatic populations of different species, and in particular, red-colored males frequently dominate over differently colored opponents (Barlow 1973; Dijkstra et al. 2005; Pryke and Griffith 2006; Healey et al. 2007; Ninnes and Andersson 2014). We show that dominance of red males can also occur in encounters of allopatric color morphs, as red *Tropheus* males prevailed over bluish and over yellow-blotch males in dyadic contests for territories. The advantage of red males could be due to an intimidating effect of their red coloration on opponent males or due to increased aggression levels, and specific experiments, for example, masking red coloration need to be carried out to distinguish between these possibilities. In the present study, the behavior of bluish males, which avoided to challenge red males and readily gave up their territorial claims in the contest trials, suggested that they were intimidated by the color or the behavior of their red opponents. A receiver bias for red agonistic signaling has recently been demonstrated in a songbird species lacking red signals (Ninnes and Andersson 2014) and is one possible explanation for the reactions of non-red *Tropheus* to red opponents.

Mate choice experiments with red-morph *Tropheus* (Hermann et al. 2015) and field observations in the geographically and genetically distant *Tropheus* sp. “black” from Bemba (Yanagisawa and Nishida 1991) demonstrated a strong influence of male territory characteristics on female mate preferences. Therefore, differential territorial success of color morphs in male–male competition may also influence mating patterns among morphs. Although the mate choice ponds were furnished with one rock cairn for each stocked male, male–male competition for cairns (i.e., territories) likely arose when highly competitive individuals claimed more than one cairn, and females, which defend their own territories when not mated, also occupied some cairns. The frequencies of positive and negative assortative mating in the mate choice ponds were consistent with interactions between red male dominance and known female preferences (Table1). While dominance of red males is expected to reinforce the positive assortative preferences of red females observed in two-way choice experiments (Egger et al. 2008), it can at the same time interfere with assortative mating of both bluish and yellow-blotch females. Indeed, the proportion of bluish females discriminating against red males dropped from 86% in the two-way choice experiment (Egger et al. 2008) to 65% in the R-B ponds (Table1).

Hence, by encouraging positive assortative mating by females of one taxon and negative assortative mating by females of the other taxon, asymmetric competitive abilities can fuel asymmetric hybridization (Rosenfield and Kodric-Brown 2003; Jones et al. 2008; Grava et al. 2012a). In particular, color-dependent dominance may influence pair formation in polymorphic species such as Gouldian finches and Midas cichlids, in which orange- or red-colored males dominate over other males (Barlow 1983; Pryke and Griffith 2006). In both species, mating is predominantly assortative by color, but mixed pairs occur and are more often composed of orange/red-colored males mated to females of the other color morph than vice versa (Pryke and Griffith 2007; Elmer et al. 2009). In contrast to the above examples, asymmetries in competitive abilities can also launch processes which promote assortative mating, for example when asymmetric dominance relationships between taxa underlie their segregation into different habitats (Vallin and Qvarnström 2011; Vallin et al., 2012b; Winkelmann et al. 2014). We cannot rule out the possibility that asymmetric dominance relationships between *Tropheus* morphs in secondary contact would also result in habitat segregation and thereby strengthen rather than weaken reproductive isolation. Segregation by depth occurred in an area of sympatry of distinct *Tropheus* species, where *T. polli* is constrained to the very shallow littoral and *T. *sp. “black” is most abundant in depths between 3 and 5 m (Kerschbaumer et al. 2014). However, in contrast to the closely related color morphs within *T. moorii*, which were investigated in the present study, *T. polli* and *T. *sp. “black” may already have been reproductively isolated at the time of coming into secondary contact, as their divergence predates major lake-level fluctuations which shaped the current distribution of littoral populations (Koblmüller et al. 2010). The space along which *Tropheus* populations can segregate by depth is defined by the range of algal growth and extends approximately 10–40 m in width, depending on the slope of the shore. Spatial segregation by depth may therefore be on a too small scale to prohibit interbreeding in the face of incomplete premating isolation. Indeed, genetic analyses revealed several examples of *Tropheus* populations with mixed genetic origin, suggesting that secondary contact in the course of lake-level fluctuations has frequently led to introgression between genetically differentiated lineages (Egger et al. 2007). In particular, the genetically distinct color morphs investigated in the present study are separated by populations, which carry genetic signatures of ancient admixture between the adjacent color morphs (Fig.1; Sturmbauer et al. 2005; Egger et al. 2007; Sefc et al. 2007; Mattersdorfer et al. 2012). Mating preferences in the bluish morph may have further evolved since last contact, but our experiments predict that male–male competition would still interfere with premating isolation in renewed contact.

### Asymmetric reproductive isolation, but no color morph-dependent dominance

Carotenoid-based ornaments, such as the blotches on the flanks of the yellow-blotch *Tropheus* (Mattersdorfer et al. 2012), are frequently correlated with reproductive and competitive success (Svensson and Wong 2011; Sefc et al. 2014), but apparently, this is not the case when the yellow-blotch males compete for mates and territories with the bluish males, which lack such ornaments. Preferences of yellow-blotch females shown in two-way choice experiments with bluish males had been interpreted as random with respect to male color morph (Egger et al. 2010; Table1), whereas parentage in the present experiment (YB-B) revealed a significant excess of negative assortative mating, which did not coincide with asymmetries in male competitive abilities in staged contests (Table1). Yellow-blotch males had offspring with bluish, but not with yellow-blotch females in two ponds (Fig.2C, Table S4), which indicates that yellow-blotch males were in principle available for mating in these ponds, but were not selected by yellow-blotch females. The factors responsible for the mating patterns between yellow-blotch and bluish *Tropheus* are still elusive. Yellow-blotch and bluish morphs are more closely related to each other than both are to the red morph (Fig.1). If the time since divergence between the yellow-blotch and the bluish lineage was too short to allow the evolution of positive assortative preferences, we would expect random mate choice by females of one or both morphs. In contrast, both tested populations deviated from random mating by displaying either significantly positive (bluish females) or significantly negative (yellow-blotch females) preferences. As yellow-blotch females had been observed to actively court foreign males in previous two-way choice experiments (Egger et al. 2010; Zoppoth et al. 2013), the negative assortative mating by yellow-blotch females in the ponds likely resulted from female preferences for bluish males. Hence, our findings indicate that mate choice of yellow-blotch females is apparently not determined by sexual imprinting on the parental phenotype. Across populations, the parentage data of the current study confirm the existence of substantial differences in the degrees of assortative mating preferences among color morphs (Egger et al. 2010), suggesting that the mechanisms underlying mating preferences evolved in different ways in these young lineages.

### Inference of mate choice from parentage in pond experiments: potential caveats

In the present study, we used genetic parentage analysis to infer the numbers of matings and hence mate choice between color morphs. Before interpreting our results, we discuss the potential caveats of this approach and why we consider it valid for the tested *Tropheus* morphs. Theoretically, the availability of unmated males could influence female mate choice beyond the effects of male–male competition and female preferences, especially given that ponds were stocked with a surplus of females. However, males invest considerably less time in reproduction than the mouthbrooding females (Yanagisawa and Sato 1990; Sefc 2008), which should allow females a choice of unmated males throughout the experiment. Furthermore, discrepancies between mate choice and paternity could arise from alternative male reproductive tactics. Sneaking and other types of reproductive parasitism were never detected in nature, where *Tropheus* also occur at the high densities conducive to these behaviors. For instance, wild-caught *Tropheus* broods were found to be sired by a single male each (Egger et al. 2006). Therefore, we consider it unlikely that alternative tactics of pond males compromised our inferences on mate choice and assume that maternal half sibs arose from independent breeding events. Support for this assumption comes from the observation that in the R-YB experiment, which had been run for a shorter time (6 months) than the other two experiments (1 year) and therefore allowed for fewer mating events per female (Yanagisawa and Nishida 1991), females had offspring with no more than one male each.

Finally, reduced survival of heteromorphic offspring could affect the inference of mate choice from sampled offspring. However, reciprocal crosses of bluish × yellow-blotch and bluish × red *Tropheus* in no-choice conditions produced similar numbers of offspring as do crosses between homomorphic parents (C. Sturmbauer and K. Sefc; pers. obs.). Furthermore, the numbers of sampled offspring per parent pair (representing brood sizes) in the present experiments did not differ between heteromorphic and homomorphic parents (mean sizes of full sib groups: 6.4 and 6.2 with homo- and heteromorphic parents, respectively; *t* = 0.3058, df = 84.194, *P* = 0.7605; Tables S1–S3), nor was there a difference between parental combinations (red × red, red × bluish, etc.; ANOVA, df = 7, *F* = 1.609, *P* = 0.136). Provided that most full sibs originate from the same mating event, which is likely because of the long spawning intervals of *Tropheus* females (Yanagisawa and Nishida 1991), this suggests that hatching success and survival were not reduced in heteromorphic offspring.

### Implications of behavioral isolation estimates for species delineation

The systematic classification of allopatric taxa is notoriously difficult (Genner et al. 2004). In *Tropheus*, the large color pattern diversity among allopatric populations contrasts with the formal description of only four nominal species (plus two junior synonyms; Konings 2013). The type specimens of *T. moorii* belong to either the yellow-blotch or, according to new investigations, the bluish morph (Konings 2013). Close genetic relatedness (Fig.1) and incomplete reproductive isolation (current study) between the two color morphs clearly justify the classification of both yellow-blotch and bluish *Tropheus* as *T. moorii*.

The red morph, which appears closely related to the yellow-blotch and bluish morph in a nuclear marker tree (Fig.1), was suggested to merit species status (*Tropheus* sp. “red”, Konings 1998). This suggestion is founded on the observation that at locations north of the lake section shown in Fig.1, red *Tropheus* occur in sympatry with differently colored congeners (*Tropheus* color type Murago), which are currently assigned to *T. moorii* (Konings 1998; van Steenberge et al. 2011). However, the Murago-type populations are genetically (Fig.1; Egger et al. 2007) and morphologically (M. van Steenberge, pers. comm.) distinct from the southern (bluish and yellow-blotch) *T. moorii* and likely represent a separate species (M. van Steenberge, unpublished). Consequently, sympatry of the red *Tropheus* morph with Murago-type *Tropheus* would not preclude the assignment of the red morph to *T. moorii*. Considering the rates of introgression between the yellow-blotch or bluish *T. moorii* and the red *Tropheus* both in the current experiments and in anthropogenic admixture (Salzburger et al. 2006; Egger et al. 2012), the retention of the three color morphs within the same species, *T. moorii*, seems indicated.

## Conclusions

The evolution of mating preferences during allopatric divergence apparently does not follow the same trajectory in the investigated populations. Reproductive isolation was asymmetric in all three comparisons, and even though females of one population may discriminate strongly against foreign males, population admixture will be initiated by the less discriminating females of the other population.

Moreover, our data support a role for male–male competition in determining the strength and pattern of assortative mating between taxa. In particular, a competitive advantage of one population over the other can reduce premating isolation by provoking asymmetric introgression and consequently interfere with speciation. A negative effect of asymmetric dominance on premating isolation is especially likely in environments, which do not support sufficient spatial segregation by competition-induced divergence in habitat use.

Competitive advantages are sometimes linked to red coloration. Red male dominance in contests between allopatric taxa, as shown here, implies that competitive interactions between color morphs in secondary contact can immediately be affected by asymmetric dominance, without requiring a prolonged period of sympatry to learn or evolve the underlying mechanism.

## Supporting Information

**Figure S1.** Tank setup in competition trials with the red and bluish color morph (R-B experiment).

**Table S1.** Outcomes of staged territorial contests (R-B), and weight of contestants.

**Table S2.** Parentage assignment in the experiment with bluish (Chaitika) and red (Chimba) *Tropheus*.

**Table S3.** Outcomes of staged territorial contests (R-YB), and weight and size (standard length SL) of contestants.

**Table S4.** Parentage assignment in the experiment with red (Chimba) and yellow-blotch (Mbita) *Tropheus*.

**Table S5.** Outcomes of staged territorial contests (YB-B), and weight and size (standard length SL) of contestants.

**Table S6.** Parentage assignment in the experiment with bluish (Funda) and yellow-blotch (Mbita) *Tropheus*.
