# Supplementary material for: Asymmetric dominance and asymmetric mate choice oppose premating isolation after allopatric divergence
Source: Ecol Evol. 2015 Mar 13;5(8):1549–62. doi: 10.1002/ece3.1372 (PMC4409405; doi:10.1002/ece3.1372)

Supplementary Figure S1: Tank setup in competition trials with the red and bluish color morph (R-B experiment). Contests were watched by the experimenter, and the trials were terminated immediately when the subordinate fish was identified.


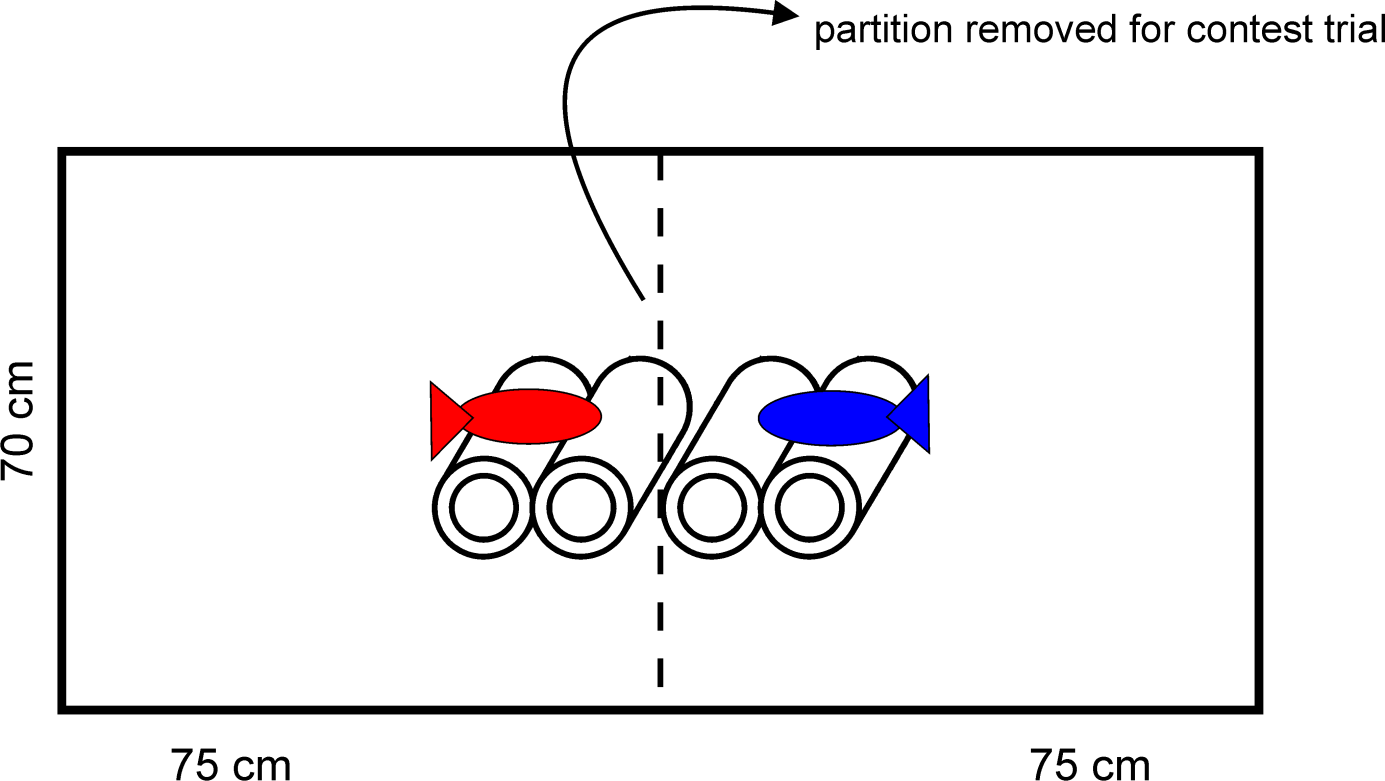

Supplement: Supplementary file 1 [file ece30005-1549-sd1.docx]
