# Supplementary material for: Asymmetric dominance and asymmetric mate choice oppose premating isolation after allopatric divergence
Source: Ecol Evol. 2015 Mar 13;5(8):1549–62. doi: 10.1002/ece3.1372 (PMC4409405; doi:10.1002/ece3.1372)
Supplement: Supplementary file 2 [file ece30005-1549-sd2.docx]

Table S1: Outcomes of staged territorial contests (R-B), and weight of contestants.

R: red morph, B: bluish morph

| **R male** | **R male weight (g)** | **B male** | **B male weight (g)** | **Winner** |
| --- | --- | --- | --- | --- |
| MM02 | 18 | NM06 | 18 | R |
| MM05 | 18 | NM11 | 21 | B |
| MM06 | 20 | CM01 | 28 | R |
| MM12 | 22 | NM05 | 18 | R |
| MM01 | 22 | NM12 | 16 | R |
| MM04 | 23 | NM03 | 19 | R |
| MM08 | 28 | NM04 | 20 | B |
| MM02 | 18 | NM04 | 19 | R |
| MM05 | 18 | NM06 | 19 | R |
| MM06 | 21 | NM11 | 22 | R |
| MM12 | 24 | CM01 | 27 | R |
| MM01 | 22 | NM05 | 18 | R |
| MM04 | 21 | NM12 | 16 | R |
| MM08 | 28 | NM03 | 20 | R |
| MM02 | 18 | NM03 | 19 | R |
| MM05 | 18 | NM04 | 21 | R |
| MM06 | 21 | NM06 | 19 | R |
| MM12 | 23 | NM11 | 22 | R |
| MM01 | 22 | CM01 | 28 | R |
| MM04 | 22 | NM05 | 19 | R |
| MM08 | 27 | NM12 | 16 | R |
| MM02 | 18 | NM12 | 17 | R |
| MM05 | 18 | NM03 | 20 | R |
| MM06 | 21 | NM04 | 21 | R |
| MM12 | 24 | NM06 | 18 | R |
| MM01 | 22 | NM11 | 22 | R |
| MM04 | 22 | CM01 | 27 | R |
| MM08 | 27 | NM05 | 18 | R |
| MM02 | 18 | NM05 | 19 | R |
| MM05 | 19 | NM12 | 18 | R |
| MM06 | 22 | NM03 | 19 | R |
| MM12 | 24 | NM04 | 22 | R |
| MM01 | 22 | NM06 | 20 | R |
| MM04 | 22 | NM11 | 23 | R |
| MM08 | 28 | CM01 | 29 | R |
| MM02 | 18 | CM01 | 29 | R |
| MM05 | 19 | NM05 | 18 | R |
| MM06 | 22 | NM12 | 18 | R |
| MM12 | 23 | NM03 | 19 | R |
| MM01 | 23 | NM04 | 22 | R |
| MM04 | 23 | NM06 | 20 | R |
| MM08 | 28 | NM11 | 23 | R |
| MM02 | 18 | NM11 | 23 | R |
| MM05 | 19 | CM01 | 29 | R |
| MM06 | 22 | NM05 | 19 | R |
| MM12 | 24 | NM12 | 18 | R |
| MM01 | 22 | NM03 | 19 | R |
| MM04 | 23 | NM04 | 22 | R |
| MM08 | 28 | NM06 | 19 | R |
