# Supplementary material for: Asymmetric dominance and asymmetric mate choice oppose premating isolation after allopatric divergence
Source: Ecol Evol. 2015 Mar 13;5(8):1549–62. doi: 10.1002/ece3.1372 (PMC4409405; doi:10.1002/ece3.1372)
Supplement: Supplementary file 3 [file ece30005-1549-sd3.docx]

Table S2. Parentage assignment in the experiment with bluish (Chaitika) and red (Chimba) *Tropheus*. For each pond, the numbers of offspring assigned to the different parent pairs are given. Stocked adults, which survived throughout the period of the experiment, are identified by bold print.

| Females |  |  |  | *T*. Chaitika males | | | | *T*. Chimba males | | | |
| --- | --- | --- | --- | --- | --- | --- | --- | --- | --- | --- | --- |
|  | Weight (g) | SL (cm) |  | Chai.m1 | **Chai.m2** | **Chai.m3** | **Chai.m4** | Chim.m1 | Chim.m2 | **Chim.m3** | **Chim.m4** |
| *Pond a* |  |  | Weight (g) | 16/- | 25/26 | 12/25 | 15/28 | 27/- | 30/- | 17/23 | 19/26 |
|  |  |  | SL (cm) | 7.2/- | 8.9/8.9 | 6.9/8.4 | 7.6/8.6 | 8.9/- | 8.5/- | 7.5/7.9 | 8.2/8.2 |
| **Chai.f1** | 16/25 | 7.7/8.1 |  |  | 5 |  |  |  |  |  |  |
| **Chai.f2** | 21/25.5 | 7.9/8.3 |  |  |  |  |  |  |  | 5 |  |
| **Chai.f3** | 17/22.5 | 7.5/7.9 |  |  | 2 |  |  |  |  |  |  |
| **Chai.f4** | 18/22 | 7.4/7.7 |  |  |  |  |  |  |  |  |  |
| **Chai.f5** | 14/21 | 7.2/7.9 |  |  |  |  |  |  |  |  |  |
| **Chai.f6** | 16/20 | 7.2/7.9 |  |  | 6 |  |  |  |  |  |  |
| Chai.f7 | 21/- | 7.9/- |  |  |  |  |  |  | 7 |  |  |
| **Chai.f8** | 24/29 | 8.5/8.7 |  |  | 6 |  |  |  |  |  |  |
| **Chim.f1** | 27/25 | 8.6/8.6 |  |  |  |  |  |  | 10 |  |  |
| **Chim.f2** | 30/25 | 8.8/8.8 |  |  |  |  |  | 9 | 8 | 2 |  |
| **Chim.f3** | 25/24.5 | 8.5/8.5 |  |  |  |  |  |  | 11 | 2 |  |
| **Chim.f4** | 35/28 | 8.7/8.7 |  |  |  |  |  |  | 3 |  |  |
| **Chim.f5** | 16/23.5 | 7.3/7.8 |  |  |  |  |  | 2 |  |  | 1 |
| **Chim.f6** | 18/22 | 7.7/8.0 |  |  |  |  |  |  | 6 | 2 |  |
| **Chim.f7** | 17/23 | 8.0/8.0 |  |  |  |  |  | 5 |  |  |  |
| **Chim.f8** | 20/21 | 7.5/7.6 |  |  |  |  |  |  | 4 | 2 |  |
|  |  |  |  | **Chai.m1** | **Chai.m2** | **Chai.m3** | Chai.m4 | **Chim.m1** | **Chim.m2** | **Chim.m3** | **Chim.m4** |
| *Pond b* |  |  | Weight (g) | 14/24 | 25/25 | 16/24 | 15/- | 21/23 | 22/26 | 22/23 | 16/24 |
|  |  |  | SL (cm) | 7.3/8.3 | 8.5/8.6 | 7.3/8.2 | 7.0/- | 8.2/8.2 | 7.4/8.3 | 8.0/8.0 | 7.0/8.1 |
| **Chai.f1** | 17/27 | 7.2/8.5 |  |  | 6 |  |  |  |  |  |  |
| **Chai.f2** | 19/24 | 7.7/8.3 |  |  | 10 |  |  |  |  |  |  |
| **Chai.f3** | 25/30 | 9.1/9.1 |  |  | 5 |  |  |  |  |  |  |
| Chai.f4 | 16/- | 7.3/- |  |  |  |  |  |  |  |  |  |
| **Chai.f5** | 19/28 | 8.1/8.7 |  |  |  |  |  |  |  |  |  |
| **Chai.f6** | 13/21.5 | 7.4/7.4 |  |  |  |  |  |  |  |  |  |
| **Chai.f7** | 20/23 | 8.4/8.4 |  |  | 8 |  |  |  |  |  |  |
| Chai.f8 | 13/- | 7.3/- |  |  |  |  |  |  |  |  |  |
| **Chim.f1** | 31/27 | 8.6/8.6 |  |  |  |  |  | 11 | 18 |  |  |
| **Chim.f2** | 24/21 | 8.1/8.1 |  |  |  |  |  | 10 |  |  |  |
| **Chim.f3** | 18/17 | 7.4/7.4 |  |  |  |  |  |  |  |  |  |
| **Chim.f4** | 22/23 | 7.9/7.9 |  |  |  |  |  | 18 |  |  |  |
| **Chim.f5** | 15/17 | 7.1/7.2 |  |  |  |  |  |  |  |  |  |
| **Chim.f6** | 30/27 | 9.1/9.1 |  |  |  |  |  | 5 | 5 |  |  |
| **Chim.f7** | 17/21.5 | 7.1/7.3 |  |  |  |  |  | 5 |  |  |  |
| **Chim.f8** | 14/21.5 | 6.6/7.3 |  |  |  |  |  |  |  |  |  |
|  |  |  |  | **Chai.m1** | Chai.m2 | **Chai.m3** | Chai.m4 | **Chim.m1** | Chim.m2 | Chim.m3 | **Chim.m4** |
| *Pond c* |  |  | Weight (g) | 20/23.5 | 18/- | 22/27 | 15/- | 28/24.5 | 19/- | 17/25 | 18/20 |
|  |  |  | SL (cm) | 7.3/8.6 | 7.3/- | 8.3/8.8 | 7.2/- | 8.5/8.6 | 7.5/- | 7.3/- | 7.8/7.8 |
| **Chai.f1** | 21/16.5 | 8.4/8.4 |  |  |  | 9 |  |  |  |  |  |
| **Chai.f2** | 18/23 | 8.0/8.0 |  |  |  | 4 |  |  |  |  |  |
| **Chai.f3** | 17/26 | 7.7/8.4 |  |  |  |  |  |  |  |  |  |
| **Chai.f4** | 16/22.5 | 7.2/8.1 |  |  |  | 14 |  |  |  |  |  |
| Chai.f5 | 20/- | 8.2/- |  |  |  | 9 |  |  |  |  |  |
| **Chai.f6** | 21/18 | 7.8/7.8 |  |  |  |  |  |  |  |  |  |
| Chai.f7 | 27/- | 8.8/- |  |  |  | 12 |  |  |  |  |  |
| Chai.f8 | 17/- | 7.6/- |  |  |  |  |  |  |  |  |  |
| **Chim.f1** | 14/17.5 | 6.9/7.0 |  |  |  |  |  |  |  |  |  |
| **Chim.f2** | 16/18 | 7.2/7.2 |  |  |  |  |  |  |  |  |  |
| Chim.f3 | 23/- | 7.4/- |  |  |  |  |  |  |  |  |  |
| **Chim.f4** | 26/22 | 8.3/8.3 |  |  |  |  |  | 17 |  |  |  |
| **Chim.f5** | 16/21 | 7.1/7.9 |  |  |  |  |  | 25 |  |  |  |
| **Chim.f6** | 15/23 | 6.9/7.8 |  |  |  |  |  |  |  |  |  |
| **Chim.f7** | 21/21 | 7.3/7.7 |  |  |  |  |  | 1 |  |  |  |
| Chim.f8 | 18/- | 7.7/- |  |  |  |  |  |  |  |  |  |
|  |  |  |  | **Chai.m1** | **Chai.m2** | Chai.m3 | **Chai.m4** | **Chim.m1** | **Chim.m2** | Chim.m3 | **Chim.m4** |
| *Pond d* |  |  | Weight (g) | 17/24.5 | 17/19 | 21/- | 14/22 | 37/30.5 | 18/24 | 15/- | 23/22.5 |
|  |  |  | SL (cm) | 7.2/8.4 | 7.2/7.6 | 8.6/- | 6.8/8.1 | 8.9/8.9 | 7.5/8.1 | 7.1/- | 8.0/8.0 |
| **Chai.f1** | 11/16 | 6.4/7.1 |  |  |  | 6 | 1 |  |  |  |  |
| **Chai.f2** | 14/19 | 6.7/6.7 |  |  |  |  |  |  |  |  |  |
| **Chai.f3** | 15/19.5 | 7.5/7.8 |  |  |  |  |  |  |  |  |  |
| **Chai.f4** | 15/20.5 | 7.4/8.2 |  |  |  |  |  |  |  |  |  |
| **Chai.f5** | 23/26.5 | 8.2/8.6 |  |  |  | 5 |  |  |  |  | 1 |
| **Chai.f6** | 13/17.5 | 6.9/7.7 |  |  |  |  |  |  |  |  | 1 |
| **Chai.f7** | 15/21 | 7.1/7.8 |  |  |  |  |  |  |  |  |  |
| **Chai.f8** | 18/19 | 7.7/7.8 |  |  |  | 9 |  |  |  |  | 6 |
| **Chim.f1** | 14/20.5 | 6.8/7.4 |  |  |  |  |  | 7 |  |  |  |
| **Chim.f2** | 24/21.5 | 8.1/8.1 |  |  |  |  |  | 7 |  |  |  |
| **Chim.f3** | 17/19.5 | 7.2/7.3 |  |  |  |  |  |  |  |  |  |
| **Chim.f4** | 14/19 | 6.4/7.2 |  |  |  |  |  | 7 |  |  |  |
| **Chim.f5** | 14/14 | 6.8/6.8 |  |  |  |  |  | 5 |  |  |  |
| **Chim.f6** | 26/20 | 8.3/8.3 |  |  |  |  |  | 18 |  |  |  |
| **Chim.f7** | 22/20.5 | 7.6/7.6 |  |  |  |  |  | 2 |  |  |  |
| **Chim.f8** | 18/19.5 | 7.2/7.4 |  |  |  |  |  | 13 |  |  |  |
|  |  |  |  | **Chai.m1** | **Chai.m2** | **Chai.m3** | **Chai.m4** | **Chim.m1** | **Chim.m2** | **Chim.m3** | **Chim.m4** |
| *Pond e* |  |  | Weight (g) | 14/23 | 14/20 | 16/22 | 14/22.5 | 27/20 | 19/24 | 27/21 | 16/21.5 |
|  |  |  | SL (cm) | 6.8/7.6 | 7.1/7.2 | 6.9/7.6 | 7.1/7.4 | 8.2/8.2 | 7.5/7.7 | 8.4/8.4 | 7.4/7.4 |
| **Chai.f1** | 26/28.5 | 8.5/8.6 |  | 6 |  |  |  | 13 |  | 10 |  |
| **Chai.f2** | 15/21 | 6.8/7.3 |  |  |  |  |  |  |  |  |  |
| **Chai.f3** | 20/24 | 7.5/7.8 |  |  |  |  |  | 2 |  |  |  |
| **Chai.f4** | 14/22.5 | 7.4/7.6 |  |  |  |  |  |  |  |  |  |
| **Chai.f5** | 17/23.5 | 7.5/7.6 |  |  |  |  |  | 5 |  | 4 |  |
| **Chai.f6** | 17/20 | 7.1/7.3 |  |  |  |  |  | 1 |  | 5 |  |
| **Chai.f7** | 14/22.5 | 7.5/7.5 |  |  |  |  |  |  |  |  |  |
| **Chai.f8** | 15/23 | 7.2/7.8 |  |  |  |  |  |  |  | 8 |  |
| **Chim.f1** | 23/20 | 7.9/7.9 |  |  |  |  |  | 9 |  |  |  |
| **Chim.f2** | 23/22 | 7.9/7.9 |  |  |  |  |  | 3 | 4 | 2 |  |
| Chim.f3 | 30/- | 9.0/- |  |  |  |  |  | 9 |  |  |  |
| **Chim.f4** | 19/18.5 | 7.4/7.4 |  |  |  |  |  |  |  |  |  |
| **Chim.f5** | 17/19 | 7.3/7.3 |  |  |  |  |  |  |  |  |  |
| **Chim.f6** | 22/22 | 7.5/7.5 |  |  |  |  |  |  |  | 5 |  |
| **Chim.f7** | 18/19.5 | 7.1/7.2 |  |  |  |  |  |  |  | 1 |  |
| **Chim.f8** | 22/20 | 7.7/7.8 |  |  |  |  |  |  |  |  |  |
