# Supplementary material for: Asymmetric dominance and asymmetric mate choice oppose premating isolation after allopatric divergence
Source: Ecol Evol. 2015 Mar 13;5(8):1549–62. doi: 10.1002/ece3.1372 (PMC4409405; doi:10.1002/ece3.1372)
Supplement: Supplementary file 4 [file ece30005-1549-sd4.docx]

Table S3: Outcomes of staged territorial contests (R-YB), and weight and size (standard length SL) of contestants.

R: red morph, YB: yellow-blotch morph

| YB male | YB male weight (g) | YB male SL (cm) | R male | R male weight (g) | R male SL (cm) | Winner |
| --- | --- | --- | --- | --- | --- | --- |
| 1_Mbita1 | 17 | 7.5 | 2_Chimba3 | 17.5 | 8.2 | R |
| 2_Mbita2 | 13 | 7.4 | 4_Chimba1 | 13 | 7.6 | R |
| 2_Mbita3 | 15.5 | 7.4 | 3_Chimba1 | 16 | 7.9 | R |
| 4_Mbita1 | 14 | 7.3 | 2_Chimba4 | 15.5 | 8 | R |
| 2_Mbita4 | 13 | 7.6 | 4_Chimba1 | 13 | 7.6 | R |
| 4_Mbita3 | 12 | 7.3 | 2_Chimba4 | 15.5 | 8 | R |
| 3_Mbita3 | 11.5 | 7.3 | 4_Chimba2 | 14 | 7.8 | R |
| 1_Mbita1 | 17 | 7.5 | 3_Chimba1 | 16 | 7.9 | R |
| 1_Mbita2 | 21 | 8 | 2_Chimba2 | 19 | 8.5 | R |
| 1_Mbita3 | 20 | 8.5 | 4_Chimba3 | 23.5 | 8.2 | R |
| 2_Mbita3 | 15.5 | 7.4 | 1_Chimba3 | 18 | 8.1 | R |
| 2_Mbita1 | 14 | 7.8 | 3_Chimba1 | 16 | 7.9 | R |
| 4_Mbita2 | 15 | 7.9 | 2_Chimba3 | 17.5 | 8.2 | R |
| 3_Mbita1 | 11 | 7.5 | 4_Chimba2 | 14 | 7.8 | R |
| 3_Mbita2 | 15.5 | 7.6 | 2_Chimba4 | 15.5 | 8 | R |
| 1_Mbita1 | 17 | 7.5 | 2_Chimba4 | 15.5 | 8 | R |
| 1_Mbita3 | 20 | 8.5 | 2_Chimba1 | 18.5 | 7.7 | R |
| 2_Mbita1 | 14 | 7.8 | 4_Chimba1 | 13 | 7.6 | R |
| 1_Mbita3 | 20 | 8.5 | 3_Chimba1 | 16 | 7.9 | R |
| 2_Mbita3 | 15.5 | 7.4 | 4_Chimba1 | 13 | 7.6 | R |
| 1_Mbita2 | 21 | 8 | 3_Chimba1 | 16 | 7.9 | R |
| 1_Mbita3 | 20 | 8.5 | 2_Chimba4 | 15.5 | 8 | R |
| 1_Mbita1 | 17 | 7.5 | 4_Chimba1 | 13 | 7.6 | R |
| 1_Mbita3 | 20 | 8.5 | 3_Chimba1 | 16 | 7.9 | R |
| 2_Mbita1 | 14 | 7.8 | 4_Chimba2 | 14 | 7.8 | YB |
| 1_Mbita1 | 17 | 7.5 | 2_Chimba1 | 18.5 | 7.7 | YB |
| 4_Mbita4 | 13 | 7.4 | 1_Chimba1 | 16 | 7.8 | YB |
| 1_Mbita3 | 20 | 8.5 | 2_Chimba3 | 17.5 | 8.2 | YB |
| 1_Mbita2 | 21 | 8 | 2_Chimba3 | 17.5 | 8.2 | YB |
| 3_Mbita2 | 15.5 | 7.6 | 4_Chimba2 | 14 | 7.8 | YB |
| 1_Mbita1 | 17 | 7.5 | 4_Chimba2 | 14 | 7.8 | YB |
