# Supplementary material for: Asymmetric dominance and asymmetric mate choice oppose premating isolation after allopatric divergence
Source: Ecol Evol. 2015 Mar 13;5(8):1549–62. doi: 10.1002/ece3.1372 (PMC4409405; doi:10.1002/ece3.1372)
Supplement: Supplementary file 5 [file ece30005-1549-sd5.docx]

Table S4. Parentage assignment in the experiment with red (Chimba) and yellow-blotch (Mbita) *Tropheus*. For each pond, the numbers of offspring assigned to the different parent pairs are given. Stocked adults, which survived throughout the period of the experiment, are identified by bold print.

| **Females** |  |  |  | *T*. Chimba males | | | | *T*. Mbita males | | | |
| --- | --- | --- | --- | --- | --- | --- | --- | --- | --- | --- | --- |
|  | Weight (g) | SL (cm) |  | Chim.m1 | **Chim.m2** | **Chim.m3** | **Chim.m4** | Mbit.m1 | **Mbit.m2** | **Mbit.m3** | **Mbit.m4** |
| ***Pond a*** |  |  | Weight (g) |  | 18 | 23 | 16 |  | 20 | 17 | 21 |
|  |  |  | SL (cm) |  | 8.1 | 8.2 | 7.8 |  | 8.5 | 7.5 | 8.0 |
| **Chim.f1** | 11.5 | 7.1 |  |  |  |  |  |  |  |  |  |
| Chim.f2 |  |  |  |  |  |  |  |  |  |  |  |
| Chim.f3 |  |  |  |  |  |  |  |  |  |  |  |
| **Chim.f4** | 15 | 7.4 |  |  | 7 |  |  |  |  |  |  |
| **Chim.f5** | 12 | 6.8 |  |  | 11 |  |  |  |  |  |  |
| **Chim.f6** | 13 | 7.4 |  |  | 14 |  |  |  |  |  |  |
| **Chim.f7** | 11 | 7.0 |  |  | 8 |  |  |  |  |  |  |
| Chim.f8 |  |  |  |  |  |  |  |  |  |  |  |
| **Mbit.f1** | 12.5 | 7.3 |  |  |  |  |  |  | 4 |  |  |
| **Mbit.f2** | 14 | 7.8 |  |  |  |  |  |  | 2 |  |  |
| **Mbit.f3** | 15 | 7.6 |  |  |  |  |  |  | 8 |  |  |
| **Mbit.f4** | 15.5 | 7.6 |  |  |  |  |  |  |  |  |  |
| **Mbit.f5** | 11 | 6.9 |  |  |  |  |  |  | 5 |  |  |
| **Mbit.f6** | 14.5 | 7.6 |  |  |  |  |  |  | 16 |  |  |
| **Mbit.f7** | 15 | 7.4 |  |  |  |  |  |  |  |  |  |
| **Mbit.f8** | 8 | 6.7 |  |  |  |  |  |  |  |  |  |
|  |  |  |  | **Chim.m1** | **Chim.m2** | **Chim.m3** | **Chim.m4** | **Mbit.m1** | **Mbit.m2** | **Mbit.m3** | **Mbit.m4** |
| ***Pond b*** |  |  | Weight (g) | 15.5 | 17.5 | 19 | 18.5 | 13 | 13 | 18 | 15.5 |
|  |  |  | SL (cm) | 8.0 | 8.2 | 8.5 | 7.7 | 7.4 | 7.6 | 8.0 | 7.4 |
| **Chim.f1** | 7.5 | 6.4 |  |  |  |  |  |  |  |  |  |
| **Chim.f2** | 8 | 6.4 |  |  |  |  |  |  |  |  |  |
| **Chim.f3** | 8.5 | 6.6 |  |  | 2 |  |  |  |  |  |  |
| **Chim.f4** | 11 | 6.8 |  |  |  |  |  |  |  |  |  |
| Chim.f5 |  |  |  |  |  |  |  |  |  |  |  |
| **Chim.f6** | 10 | 6.7 |  |  |  |  |  |  |  |  |  |
| **Chim.f7** | 7 | 6.5 |  |  |  |  |  |  |  |  |  |
| **Chim.f8** | 8 | 6.5 |  |  |  |  |  |  |  |  |  |
| **Mbit.f1** | 13.5 | 7.6 |  |  |  | 7 |  |  |  |  |  |
| **Mbit.f2** | 28 | 8.2 |  |  |  | 9 |  |  |  |  |  |
| **Mbit.f3** | 14.5 | 7.5 |  |  |  | 7 |  |  |  |  |  |
| **Mbit.f4** | 17 | 8.0 |  |  |  |  |  |  |  |  |  |
| **Mbit.f5** | 12 | 7.1 |  |  |  |  |  |  |  |  |  |
| **Mbit.f6** | 14 | 7.8 |  |  |  |  |  |  |  |  |  |
| **Mbit.f7** | 15.5 | 7.6 |  |  |  |  |  |  |  |  |  |
| **Mbit.f8** | 11 | 7.3 |  |  |  |  |  |  |  |  |  |
|  |  |  |  | Chim.m1 | **Chim.m2** | ^a)^ | ^a)^ | **Mbit.m1** | Mbit.m2 | **Mbit.m3** | **Mbit.m4** |
| ***Pond c*** |  |  | Weight (g) |  | 16 |  |  | 15.5 |  | 11.5 | 11 |
|  |  |  | SL (cm) |  | 7.9 |  |  | 7.6 |  | 7.3 | 7.5 |
| **Chim.f1** | 13 | 7.7 |  |  |  |  |  |  |  |  |  |
| **Chim.f2** | 12 | 7.6 |  |  |  |  |  |  |  |  |  |
| **Chim.f3** | 15 | 8.1 |  |  | 11 |  |  |  |  |  |  |
| **Chim.f4** | 18 | 8.1 |  |  | 9 |  |  |  |  |  |  |
| **Chim.f5** | 16.5 | 7.9 |  |  |  |  |  |  |  |  |  |
| **Chim.f6** | 11.5 | 8.0 |  |  |  |  |  |  |  |  |  |
| **Chim.f7** | 14 | 7.5 |  |  |  |  |  |  |  |  |  |
| **Chim.f8** | 17.5 | 8 |  |  |  |  |  |  |  |  |  |
| **Chim.f9** ^a)^ | 16 | 7.7 |  |  | 7 |  |  |  |  |  |  |
| **Chim.f10** ^a)^ | 16 | 7.8 |  |  |  |  |  |  |  |  |  |
| **Mbit.f1** | 9.5 | 6.8 |  |  |  |  |  |  |  |  |  |
| **Mbit.f2** | 8.5 | 6.2 |  |  |  |  |  |  |  |  |  |
| **Mbit.f3** | 9 | 6.9 |  |  |  |  |  |  |  |  |  |
| **Mbit.f4** | 9 | 6.4 |  |  |  |  |  |  |  |  |  |
| **Mbit.f5** | 13 | 7.4 |  |  | 10 |  |  |  |  |  |  |
| **Mbit.f6** | 8 | 6.7 |  |  |  |  |  |  |  |  |  |
| Mbit.f7 |  |  |  |  |  |  |  |  |  |  |  |
| **Mbit.f8** | 13 | 7.5 |  |  |  |  |  |  |  |  |  |
|  |  |  |  | **Chim.m1** | **Chim.m2** | Chim.m3 | **Chim.m4** | **Mbit.m1** | **Mbit.m2** | **Mbit.m3** | **Mbit.m4** |
| ***Pond d*** |  |  | Weight (g) | 23.5 | 13 |  | 14 | 15 | 12 | 14 | 13 |
|  |  |  | SL (cm) | 8.2 | 7.6 |  | 7.8 | 7.9 | 7.3 | 7.3 | 7.4 |
| **Chim.f1** | 13 | 7.2 |  |  |  |  |  |  |  |  |  |
| **Chim.f2** | 15.5 | 8.3 |  | 8 |  |  |  |  |  |  |  |
| **Chim.f3** | 16 | 7.9 |  |  |  |  |  |  |  |  |  |
| **Chim.f4** | 12.5 | 7.3 |  | 7 |  |  |  |  |  |  |  |
| Chim.f5 |  |  |  |  |  |  |  |  |  |  |  |
| **Chim.f6** | 16.5 | 7.5 |  | 6 |  |  |  |  |  |  |  |
| **Chim.f7** | 18 | 8.1 |  | 8 |  |  |  |  |  |  |  |
| **Chim.f8** | 15 | 7.0 |  | 7 |  |  |  |  |  |  |  |
| **Mbit.f1** | 8 | 7.0 |  |  |  |  |  | 4 |  |  |  |
| **Mbit.f2** | 11 | 6.7 |  |  |  |  |  |  |  |  |  |
| **Mbit.f3** | 8 | 6.0 |  |  |  |  |  |  |  |  |  |
| **Mbit.f4** | 9.5 | 6.4 |  |  |  |  |  |  |  |  |  |
| **Mbit.f5** | 11 | 6.4 |  |  |  |  |  |  |  |  |  |
| **Mbit.f6** | 9 | 6.3 |  |  |  |  |  |  |  |  |  |
| **Mbit.f7** | 14.5 | 7.4 |  |  |  |  |  |  |  |  |  |
| **Mbit.f8** | 8 | 6.7 |  |  |  |  |  |  |  |  |  |

^a)^ In pond c, two individuals stocked as males (Chimba) were recognized as females at the termination of the experiment. False sexing can occur in Tropheus when the genital papilla – the trait used to distinguish males from females – is not well developed in young mature females.
