# Supplementary material for: Asymmetric dominance and asymmetric mate choice oppose premating isolation after allopatric divergence
Source: Ecol Evol. 2015 Mar 13;5(8):1549–62. doi: 10.1002/ece3.1372 (PMC4409405; doi:10.1002/ece3.1372)
Supplement: Supplementary file 6 [file ece30005-1549-sd6.docx]

Table S5: Outcomes of staged territorial contests (YB-B), and weight and size (standard length SL) of contestants.

YB: yellow-blotch morph, B: bluish morph

| YB male | YB male weight (g) | YB male SL (cm) | B male | B male weight (g) | B male SL (cm) | Winner |
| --- | --- | --- | --- | --- | --- | --- |
| Y10c | 20 | 8.4 | B30d | 27 | 8.4 | B |
| Y10c | 20 | 8.4 | B22b | 25 | 8.6 | B |
| Y10c | 20 | 8.4 | B29d | 29 | 8.6 | B |
| Y10c | 20 | 8.4 | B31d | 29 | 8.7 | B |
| Y10c | 20 | 8.4 | B32d | 31 | 8.9 | B |
| Y10c | 20 | 8.4 | B18a | 29 | 9.5 | B |
| Y11c | 25 | 8.9 | B32d | 31 | 8.9 | B |
| Y11c | 25 | 8.9 | B31d | 29 | 8.7 | B |
| Y11c | 25 | 8.9 | B17a | 29 | 9.2 | B |
| Y11c | 25 | 8.9 | B30d | 27 | 8.4 | B |
| Y11c | 25 | 8.9 | B18a | 29 | 9.5 | B |
| Y12c | 20 | 7.4 | B21b | 18 | 8 | B |
| Y12c | 20 | 7.4 | B30d | 27 | 8.4 | B |
| Y12c | 20 | 7.4 | B31d | 29 | 8.7 | B |
| Y12c | 20 | 7.4 | B32d | 31 | 8.9 | B |
| Y12c | 20 | 7.4 | B17a | 29 | 9.2 | B |
| Y12c | 20 | 7.4 | B18a | 29 | 9.5 | B |
| Y13d | 25 | 8.3 | B22b | 25 | 8.6 | B |
| Y13d | 25 | 8.3 | B17a | 29 | 9.2 | B |
| Y13d | 25 | 8.3 | B18a | 29 | 9.5 | B |
| Y14d | 23 | 8.2 | B23b | 16 | 8.1 | B |
| Y14d | 23 | 8.2 | B22b | 25 | 8.6 | B |
| Y14d | 23 | 8.2 | B17a | 29 | 9.2 | B |
| Y14d | 23 | 8.2 | B18a | 29 | 9.5 | B |
| Y15d | 19 | 7.1 | B22b | 25 | 8.6 | B |
| Y15d | 19 | 7.1 | B17a | 29 | 9.2 | B |
| Y15d | 19 | 7.1 | B18a | 29 | 9.5 | B |
| Y16d | 18 | 7.4 | B26c | 19 | 8.4 | B |
| Y16d | 18 | 7.4 | B22b | 25 | 8.6 | B |
| Y16d | 18 | 7.4 | B27c | 27 | 8.8 | B |
| Y16d | 18 | 7.4 | B17a | 29 | 9.2 | B |
| Y16d | 18 | 7.4 | B18a | 29 | 9.5 | B |
| Y1a | 31 | 9.7 | B31d | 29 | 8.7 | B |
| Y2a | 29 | 9.4 | B31d | 29 | 8.7 | B |
| Y5b | 26 | 8.2 | B30d | 27 | 8.4 | B |
| Y5b | 26 | 8.2 | B29d | 29 | 8.6 | B |
| Y5b | 26 | 8.2 | B31d | 29 | 8.7 | B |
| Y5b | 26 | 8.2 | B25c | 30 | 8.8 | B |
| Y5b | 26 | 8.2 | B32d | 31 | 8.9 | B |
| Y5b | 26 | 8.2 | B18a | 29 | 9.5 | B |
| Y9c | 22 | 8.4 | B30d | 27 | 8.4 | B |
| Y9c | 22 | 8.4 | B29d | 29 | 8.6 | B |
| Y9c | 22 | 8.4 | B31d | 29 | 8.7 | B |
| Y9c | 22 | 8.4 | B32d | 31 | 8.9 | B |
| Y9c | 22 | 8.4 | B17a | 29 | 9.2 | B |
| Y9c | 22 | 8.4 | B18a | 29 | 9.5 | B |
| Y10c | 20 | 8.4 | B23b | 16 | 8.1 | Y |
| Y10c | 20 | 8.4 | B21b | 18 | 8 | Y |
| Y11c | 25 | 8.9 | B22b | 25 | 8.6 | Y |
| Y11c | 25 | 8.9 | B29d | 29 | 8.6 | Y |
| Y11c | 25 | 8.9 | B23b | 16 | 8.1 | Y |
| Y11c | 25 | 8.9 | B21b | 18 | 8 | Y |
| Y12c | 20 | 7.4 | B23b | 16 | 8.1 | Y |
| Y13d | 25 | 8.3 | B26c | 19 | 8.4 | Y |
| Y13d | 25 | 8.3 | B21b | 18 | 8 | Y |
| Y13d | 25 | 8.3 | B25c | 30 | 8.8 | Y |
| Y13d | 25 | 8.3 | B27c | 27 | 8.8 | Y |
| Y14d | 23 | 8.2 | B21b | 18 | 8 | Y |
| Y14d | 23 | 8.2 | B26c | 19 | 8.4 | Y |
| Y14d | 23 | 8.2 | B25c | 30 | 8.8 | Y |
| Y14d | 23 | 8.2 | B27c | 27 | 8.8 | Y |
| Y15d | 19 | 7.1 | B21b | 18 | 8 | Y |
| Y15d | 19 | 7.1 | B23b | 16 | 8.1 | Y |
| Y15d | 19 | 7.1 | B27c | 27 | 8.8 | Y |
| Y16d | 18 | 7.4 | B21b | 18 | 8 | Y |
| Y16d | 18 | 7.4 | B23b | 16 | 8.1 | Y |
| Y1a | 31 | 9.7 | B32d | 31 | 8.9 | Y |
| Y1a | 31 | 9.7 | B27c | 27 | 8.8 | Y |
| Y1a | 31 | 9.7 | B22b | 25 | 8.6 | Y |
| Y1a | 31 | 9.7 | B29d | 29 | 8.6 | Y |
| Y1a | 31 | 9.7 | B30d | 27 | 8.4 | Y |
| Y1a | 31 | 9.7 | B23b | 16 | 8.1 | Y |
| Y1a | 31 | 9.7 | B21b | 18 | 8 | Y |
| Y2a | 29 | 9.4 | B32d | 31 | 8.9 | Y |
| Y2a | 29 | 9.4 | B25c | 30 | 8.8 | Y |
| Y2a | 29 | 9.4 | B27c | 27 | 8.8 | Y |
| Y2a | 29 | 9.4 | B22b | 25 | 8.6 | Y |
| Y2a | 29 | 9.4 | B30d | 27 | 8.4 | Y |
| Y2a | 29 | 9.4 | B23b | 16 | 8.1 | Y |
| Y2a | 29 | 9.4 | B21b | 18 | 8 | Y |
| Y5b | 26 | 8.2 | B26c | 19 | 8.4 | Y |
| Y5b | 26 | 8.2 | B27c | 27 | 8.8 | Y |
| Y9c | 22 | 8.4 | B23b | 16 | 8.1 | Y |
| Y9c | 22 | 8.4 | B21b | 18 | 8 | Y |
