# Supplementary material for: Asymmetric dominance and asymmetric mate choice oppose premating isolation after allopatric divergence
Source: Ecol Evol. 2015 Mar 13;5(8):1549–62. doi: 10.1002/ece3.1372 (PMC4409405; doi:10.1002/ece3.1372)
Supplement: Supplementary file 7 [file ece30005-1549-sd7.docx]

Table S6. Parentage assignment in the experiment with bluish (Funda) and yellow-blotch (Mbita) *Tropheus*. For each pond, the numbers of offspring assigned to the different parent pairs are given. Stocked adults, which survived throughout the period of the experiment, are identified by bold print.

| Females |  |  |  | *T*. Funda males | | | | *T*. Mbita males | | | |
| --- | --- | --- | --- | --- | --- | --- | --- | --- | --- | --- | --- |
|  | Weight (g) | SL (cm) |  | Fund.m1 | **Fund.m2** | **Fund.m3** | Fund.m4 | **Mbit.m1** | **Mbit.m2** | Mbit.m3 | Mbit.m4 |
| *Pond a* |  |  | Weight (g) |  | 29 | 29 |  | 31 | 29 |  |  |
|  |  |  | SL (cm) |  | 9.5 | 9.2 |  | 9.7 | 9.4 |  |  |
| Fund.f1 |  |  |  |  |  |  |  |  |  |  |  |
| **Fund.f2** | 15 | 7.8 |  |  | 1 |  |  |  |  |  |  |
| Fund.f3 |  |  |  |  |  |  |  |  |  |  |  |
| **Fund.f4** | 18 | 8.4 |  |  |  | 8 |  |  | 8 |  |  |
| **Fund.f5** | 21 | 8.4 |  |  | 3 | 8 |  |  | 2 |  |  |
| Fund.f6 |  |  |  |  |  |  |  |  |  |  |  |
| **Fund.f7** | 18 | 8.1 |  |  |  | 6 |  |  | 3 |  |  |
| **Fund.f8** | 21 | 7.8 |  |  |  | 5 |  |  |  |  |  |
| **Mbit.f1** | 30 | 10.0 |  |  |  | 18 |  | 4 |  |  |  |
| **Mbit.f2** | 25 | 9.4 |  |  |  |  |  | 7 | 4 |  |  |
| **Mbit.f3** | 22 | 8.5 |  |  |  |  |  |  |  |  |  |
| **Mbit.f4** | 29 | 9.0 |  |  |  |  |  |  |  |  |  |
| **Mbit.f5** | 23 | 8.6 |  |  |  | 10 | 1 |  |  |  |  |
| **Mbit.f6** | 17 | 7.9 |  |  | 7 |  |  |  |  |  |  |
| **Mbit.f7** | 23 | 8.5 |  |  |  |  |  | 2 | 3 |  |  |
| **Mbit.f8** | 11 | 7.2 |  |  |  |  |  |  |  |  |  |
|  |  |  |  | **Fund.m1** | **Fund.m2** | **Fund.m3** | Fund.m4 | Mbit.m1 | Mbit.m2 | **Mbit.m3** | Mbit.m4 |
| *Pond b* |  |  | Weight (g) | 16 | 18 | 25 |  |  |  | 26 |  |
|  |  |  | SL (cm) | 8.1 | 8.0 | 8.6 |  |  |  | 8.2 |  |
| Fund.f1 |  |  |  |  |  |  |  |  |  |  |  |
| **Fund.f2** | 21 | 8.2 |  |  | 5 | 7 |  |  |  |  |  |
| **Fund.f3** | 17 | 8.1 |  |  | 4 |  |  |  |  |  |  |
| **Fund.f4** | 20 | 8.8 |  |  | 5 | 5 | 1 |  |  |  |  |
| **Fund.f5** | 23 | 8.3 |  |  |  | 9 |  |  |  | 1 |  |
| **Fund.f6** | 23 | 8.6 |  |  |  | 6 |  |  |  |  |  |
| **Fund.f7** | 20 | 8.2 |  |  | 3 | 7 |  |  |  |  |  |
| **Fund.f8** | 23 | 8.3 |  |  | 5 |  |  |  |  |  |  |
| Mbit.f1 |  |  |  |  |  |  |  |  |  |  |  |
| **Mbit.f2** | 19 | 8.2 |  |  |  | 6 | 2 |  |  |  |  |
| **Mbit.f3** | 16 | 7.4 |  |  |  |  |  |  |  |  |  |
| **Mbit.f4** | 33 | 8.9 |  |  | 1 |  | 10 |  |  |  |  |
| **Mbit.f5** | 24 | 8.9 |  |  |  |  | 3 |  |  |  |  |
| **Mbit.f6** | 21 | 8.6 |  |  | 1 | 20 |  |  |  |  |  |
| **Mbit.f7** | 17 | 8.1 |  |  |  |  |  |  |  |  |  |
| **Mbit.f8** | 14 | 7.4 |  |  |  |  |  |  |  |  |  |
|  |  |  |  | **Fund.m1** | Fund.m2 | **Fund.m3** | **Fund.m4** | **Mbit.m1** | **Mbit.m2** | **Mbit.m3** | **Mbit.m4** |
| *Pond c* |  |  | Weight (g) | 27 |  | 30 | 19 | 20 | 22 | 20 | 25 |
|  |  |  | SL (cm) | 8.8 |  | 8.8 | 8.4 | 8.4 | 8.4 | 7.4 | 8.9 |
| **Fund.f1** | 22 | 8.1 |  |  | 3 |  |  |  |  |  |  |
| **Fund.f2** | 24 | 8.4 |  |  | 10 |  |  |  |  |  |  |
| Fund.f3 |  |  |  |  | 5 |  |  |  |  |  |  |
| **Fund.f4** | 18 | 7.9 |  |  |  |  | 12 |  |  |  |  |
| **Fund.f5** | 21 | 8.1 |  |  | 4 |  | 2 |  |  |  |  |
| **Fund.f6** | 17 | 7.5 |  |  | 1 | 5 |  |  |  |  | 1 |
| **Fund.f7** | 17 | 7.3 |  |  |  |  | 5 |  |  |  |  |
| **Fund.f8** | 18 | 7.5 |  |  |  |  | 5 |  |  |  |  |
| **Mbit.f1** | 23 | 8.4 |  |  |  |  | 12 |  |  |  |  |
| **Mbit.f2** | 30 | 9.1 |  |  |  | 1 | 13 |  |  |  |  |
| **Mbit.f3** | 20 | 8.6 |  |  |  |  | 7 |  |  |  |  |
| **Mbit.f4** | 20 | 8.3 |  |  |  | 13 |  |  |  |  |  |
| **Mbit.f5** | 25 | 8.5 |  |  |  |  | 2 |  |  |  |  |
| **Mbit.f6** | 15 | 7.4 |  |  |  |  |  |  |  |  |  |
| **Mbit.f7** | 17 | 7.4 |  |  |  |  |  |  |  |  |  |
| **Mbit.f8** | 18 | 7.6 |  |  |  |  |  |  |  |  |  |
|  |  |  |  | **Fund.m1** | **Fund.m2** | **Fund.m3** | **Fund.m4** | **Mbit.m1** | **Mbit.m2** | **Mbit.m3** | **Mbit.m4** |
| *Pond d* |  |  | Weight (g) | 29 | 29 | 31 | 27 | 23 | 18 | 25 | 19 |
|  |  |  | SL (cm) | 8.7 | 8.6 | 8.9 | 8.4 | 8.2 | 7.4 | 8.3 | 7.1 |
| Fund.f1 |  |  |  | 3 | 5 | 3 |  |  |  |  |  |
| **Fund.f2** | 21 | 7.8 |  |  | 12 |  | 4 |  |  |  |  |
| Fund.f3 |  |  |  | 3 | 8 | 1 |  |  |  |  |  |
| **Fund.f4** | 22 | 7.6 |  |  | 2 | 8 | 1 |  |  |  |  |
| **Fund.f5** | 15 | 6.9 |  |  | 4 | 3 |  |  |  |  |  |
| Fund.f6 |  |  |  |  | 11 |  |  |  |  |  |  |
| Fund.f7 |  |  |  | 2 | 1 |  |  |  |  |  |  |
| **Fund.f8** | 19 | 7.8 |  | 3 | 12 |  |  |  |  |  |  |
| **Mbit.f1** | 16 | 7.2 |  |  |  |  |  |  |  |  |  |
| **Mbit.f2** | 16 | 7.0 |  |  |  |  |  |  |  |  |  |
| **Mbit.f3** | 17 | 6.9 |  |  |  |  |  |  |  |  |  |
| **Mbit.f4** | 18 | 7.4 |  |  | 1 |  |  |  |  |  |  |
| **Mbit.f5** | 20 | 8.1 |  |  |  |  |  |  |  |  |  |
| **Mbit.f6** | 17 | 7.2 |  |  | 6 |  |  |  |  |  |  |
| Mbit.f7 |  |  |  |  |  |  |  |  |  |  |  |
| **Mbit.f8** | 22 | 7.5 |  |  | 4 |  |  |  |  |  |  |
|  |  |  |  |  |  |  |  |  |  |  |  |
| *Pond e* |  |  |  | **Fund.m1** | **Fund.m2** | **Fund.m3** | **Fund.m4** | **Mbit.m1** | **Mbit.m2** | **Mbit.m3** | **Mbit.m4** |
| **Fund.f1** |  |  |  |  |  | 12 |  |  |  |  |  |
| **Fund.f2** |  |  |  |  |  | 16 |  |  |  |  |  |
| **Fund.f3** |  |  |  |  |  |  |  |  |  |  |  |
| Fund.f4 |  |  |  | 9 |  | 12 |  |  |  |  |  |
| **Fund.f5** |  |  |  |  |  | 6 |  |  |  |  |  |
| **Fund.f6** |  |  |  |  |  | 8 |  |  |  |  |  |
| **Fund.f7** |  |  |  |  |  | 4 |  |  |  |  |  |
| **Fund.f8** |  |  |  |  |  | 10 |  |  |  |  |  |
| **Mbit.f1** |  |  |  |  | 11 |  |  |  |  |  |  |
| **Mbit.f2** |  |  |  | 9 |  |  |  |  |  |  |  |
| **Mbit.f3** |  |  |  |  |  | 1 |  | 1 |  |  |  |
| **Mbit.f4** |  |  |  |  |  | 9 |  |  |  |  |  |
| **Mbit.f5** |  |  |  | 7 |  |  |  | 1 |  |  |  |
| **Mbit.f6** |  |  |  |  |  | 7 |  |  |  |  |  |
| **Mbit.f7** |  |  |  |  | 2 |  |  |  |  |  |  |
| **Mbit.f8** |  |  |  |  |  | 10 |  |  |  |  |  |
